# Effects of TNFα on Dynamic Cytosolic Ca^2 +^ and Force Responses to Muscarinic Stimulation in Airway Smooth Muscle

**DOI:** 10.3389/fphys.2021.730333

**Published:** 2021-07-30

**Authors:** Young-Soo Han, Philippe Delmotte, Gary C. Sieck

**Affiliations:** Department of Physiology and Biomedical Engineering, Mayo Clinic, Rochester, MN, United States

**Keywords:** inflammatory cytokines, dynamic Ca^2+^ sensitivity, airway smooth muscle, phase-loop plots, myosin light chain, temporal delay

## Abstract

Previously, we reported that in airway smooth muscle (ASM), the cytosolic Ca^2+^ ([Ca^2+^]_*cyt*_) and force response induced by acetyl choline (ACh) are increased by exposure to the pro-inflammatory cytokine tumor necrosis factor α (TNFα). The increase in ASM force induced by TNFα was not associated with an increase in regulatory myosin light chain (rMLC_20_) phosphorylation but was associated with an increase in contractile protein (actin and myosin) concentration and an enhancement of Ca^2+^ dependent actin polymerization. The sensitivity of ASM force generation to elevated [Ca^2+^]_*cyt*_ (Ca^2+^ sensitivity) is dynamic involving both the shorter-term canonical calmodulin-myosin light chain kinase (MLCK) signaling cascade that regulates rMLC_20_ phosphorylation and cross-bridge recruitment as well as the longer-term regulation of actin polymerization that regulates contractile unit recruitment and actin tethering to the cortical cytoskeleton. In this study, we simultaneously measured [Ca^2+^]_*cyt*_ and force responses to ACh and explored the impact of 24-h TNFα on the dynamic relationship between [Ca^2+^]_*cyt*_ and force responses. The temporal delay between the onset of [Ca^2+^]_*cyt*_ and force responses was not affected by TNFα. Similarly, the rates of rise of [Ca^2+^]_*cyt*_ and force responses were not affected by TNFα. The absence of an impact of TNFα on the short delay relationships between [Ca^2+^]_*cyt*_ and force was consistent with the absence of an effect of [Ca^2+^]_*cyt*_ and force on rMLC_20_ phosphorylation. However, the integral of the phase-loop plot of [Ca^2+^]_*cyt*_ and force increased with TNFα, consistent with an impact on actin polymerization and, contractile unit recruitment and actin tethering to the cortical cytoskeleton.

## Introduction

**FIGURE 1:**
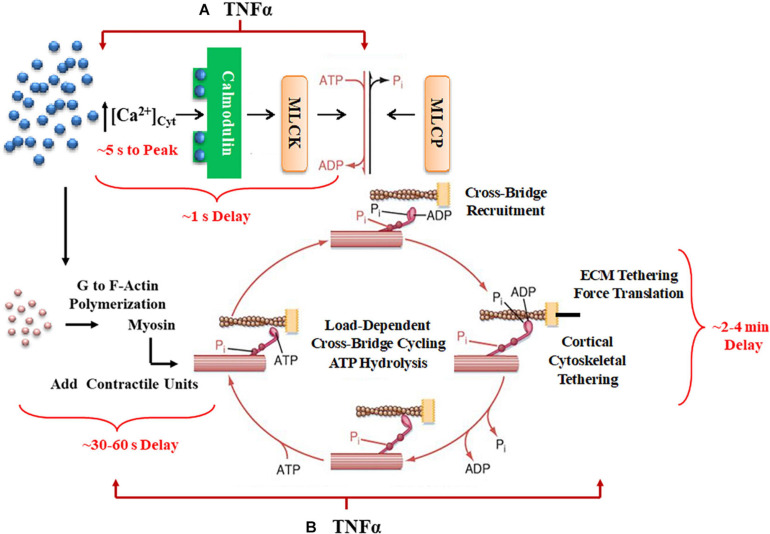
A summary schematic illustrates how TNFα would affect not only short-term time delays reflecting rMLC_20_ phosphorylation **(A)** but also would influence longer-term delays in contractile unit recruitment and internal loading in ASM. **(B)** Activation of ligand-gated or voltage-gated receptors channels by an agonist gives rise to an increase in [Ca^2+^]_*cyt*_ concentration. The increased Ca^2+^ binds to calmodulin (CaM) and then this Ca^2+^-CaM activates myosin light chain kinase (MLCK) phosphorylating rMLC_20_, which in turn allows cross-bridge (actin interaction with myosin) recruitment. When cross-bridges are formed, force is generated and transferred to the sarcolemma through the cortical cytoskeletal (actin filaments, dense bodies, and intermediate filaments) that links the contractile units to the plasma membrane. In turn, the plasma membrane of ASM cells attaches to the extracellular matrix (ECM). Cross-bridge cycling is driven by the hydrolysis of ATP. The increase in [Ca^2+^]_*cyt*_ concentration also enhances G to F-actin polymerization which contributes to the number of contractile units. Myosin light chain phosphatase (MLCP) dephosphorylates rMLC_20_, which is believed to play a role in modulating the Ca^2+^ sensitivity of force generation in ASM.

Agonists such as acetyl choline (ACh) induce a cytosolic Ca^2+^ ([Ca^2+^]_*cyt*_) and force response in airway smooth muscle (ASM). Previously, we and others showed that the regulation of [Ca^2+^]_*cyt*_ in response to ACh stimulation is dynamic as reflected by small amplitude localized [Ca^2+^]_*cyt*_ transients (Ca^2+^ sparks) that fuse into larger amplitude [Ca^2+^]_*cyt*_ oscillations that propagate through the cell and summate into larger global responses (Prakash et al., 1997; Pabelick et al., 1999; Sieck et al., 2001). As ACh stimulation increases, the amplitudes of localized [Ca^2+^]_*cyt*_ oscillations are not affected, but the frequency and propagation velocity of [Ca^2+^]_*cyt*_ oscillations increase leading to greater summation into a larger global [Ca^2+^]_*cyt*_ response in ASM cells. The coupling of [Ca^2+^]_*cyt*_ to a contractile response is also dynamic with initial force responses to elevated [Ca^2+^]_*cyt*_ being delayed by 500–800 ms in porcine ASM (Prakash et al., 1997; Pabelick et al., 1999; Sieck et al., 2001). Furthermore, a maximum steady state force response in ASM is not achieved until 2–4 min after elevation of [Ca^2+^]_*cyt*_. Thus, the dynamic sensitivity of force to elevated [Ca^2+^]_*cyt*_ is complex involving both short-term and long-term responses (Figure 1).

In smooth muscle, the initial (short-term) coupling of elevated [Ca^2+^]_*cyt*_ to a contractile response involves Ca^2+^ binding to calmodulin (CaM), Ca^2+^-CaM activating myosin light chain kinase (MLCK), which phosphorylates the regulatory myosin light chain (rMLC_20_) allowing cross-bridge recruitment and cycling (de Lanerolle and Paul, 1991; Zimmermann et al., 1995; Sieck et al., 2001; Dimopoulos et al., 2007). The level of rMLC_20_ phosphorylation is also regulated by dephosphorylation *via* MLC phosphatase (Figure 1). Generally, the sensitivity of the force response to [Ca^2+^]_*cyt*_ is thought to be influenced primarily by RhoA signaling through an effect on rMLC_20_ dephosphorylation (Zhang et al., 2012; Seow and An, 2020). The temporal delay in this canonical signaling cascade is ∼500–800 ms in porcine ASM (Sieck et al., 2001); however, the time required to reach the peak [Ca^2+^]_*cyt*_ is ∼ 5 s, and the time to reach the maximum level of rMLC_20_ phosphorylation is ∼30 s (Zhang et al., 2012).

Force generation in ASM is also affected by mobilization of *de novo* contractile units *via* polymerization of actin and myosin filaments and by internal and external loading of cross-bridges (Figure 1). In a previous study, we reported that 24-h exposure of porcine ASM to tumor necrosis factor α (TNFα) increases force generation in response to ACh stimulation by increasing the number of contractile units (actin and myosin concentration) (Dogan et al., 2017). However, TNFα exposure did not affect the extent of rMLC_20_ phosphorylation. The dynamic nature of force in ASM is also influenced by internal loading of cross-bridges due to the tethering of filamentous actin to the cortical cytoskeleton. As actin filaments are tethered, internal loading increases and cross-bridge cycling rate decreases as reflected by a slowing in the rate of ATP hydrolysis. This tethering process in ASM appears to be much slower with a steady state of ATP hydrolysis reached only after ∼5 min (Delmotte et al., 2020). Thus, the dynamic relationships between [Ca^2+^]_*cyt*_ and force responses to ACh stimulation provide insight into the influence of inflammation as mediated by TNFα.

In the present study, we simultaneously measured [Ca^2+^]_*cyt*_ and force responses to ACh stimulation in porcine ASM to determine the impact of 24-h TNFα exposure. The dynamic Ca^2+^ sensitivity of force generation in ASM was assessed using phase-loop plots of [Ca^2+^]_*cyt*_ and force responses as previously in ASM (Han et al., 2021) and cardiomyocytes (Spurgeon et al., 1992; Schaible et al., 2016, 2018; Han et al., 2018). We hypothesized that TNFα exposure does not impact short-term time delays reflecting rMLC_20_ phosphorylation but does affect longer-term delays in contractile unit recruitment and internal loading in ASM.

## Materials and Methods

### Porcine Airway Smooth Muscle Preparation

Porcine tracheas from both female and male pigs were obtained from a local abattoir, with exemption from Institutional Animal Care and Use Committee (IACUC) approval. During transport to the lab, tracheas were kept in ice-cold physiologic saline solution (composition in mM: 118.99 NaCl, 1.17 MgSO_4_, 1.18 KH_2_PO_4_, 4.7 KCl, 2.5 CaCl_2_, 0.03 EDTA, 5.5 dextrose, 25 HEPES, and pH 7.4). The ASM layer from each trachea was dissected under a binocular microscope, and eight strips (∼0.8 × 4 mM) were cut as previously described (Fredberg et al., 1996; Sieck et al., 1998, 2001, 2019; Jones et al., 1999a; Dogan et al., 2017). The ASM strips were separated into two groups: one group exposed to TNFα (100 ng/ml; Cell Sciences, MA, United States) for 24 h at room temperature and a second group exposed to physiological saline for the same period (control). Four of the ASM strips (two TNFα treated and two controls) were used for duplicate measurements of simultaneous [Ca^2+^]_*cyt*_ and force responses to ACh stimulation (EC_50_; 2.6 μM for control and 1.3 μM for TNFα). The second set of four ASM strips (two TNFα treated and two controls) were used to examine ACh-induced changes in rMLC_20_ phosphorylation.

### Simultaneous Measurements of [Ca^2+^]_*cyt*_ and Force Responses to ACh Stimulation

To measure [Ca^2+^]_*cyt*_, the ASM strips were incubated for 4 h (at 25°C) in physiological saline solution containing the acetoxymethyl ester of fura-2 (fura-2 AM; 5 μM) dissolved in dimethyl sulfoxide (DMSO) as well as 0.02% Pluronic (F-127; Molecular Probes) to facilitate efficient cytosolic loading of fura-2 AM. The ASM strips were then mounted using stainless steel microforceps between a micropositioner (Mitutoyo America Corp., Aurora, IL, United States) to adjust muscle length and an isometric force transducer (KG4; Scientific Instruments, GmbH, Heidelberg, Germany) in a 0.2-ml quartz cuvette (Guth Muscle Research System; Scientific Instruments GmbH, Heidelberg, Germany) (Guth and Wojciechowski, 1986; Sieck et al., 2019) that was continuously perfused with physiological saline solution aerated with 95% O_2_ and 5% CO_2_. The ASM strips were perfused for 30 min to wash out any excess fura-2 AM. The length of the ASM strip was then adjusted over a 1-h period to achieve a sustained preload force of 10 mN.

Fura-2 fluorescence ion the ASM strip was excited using a mercury high-pressure lamp (75 W) as a light source at alternating (every 2 ms) wavelengths of 340 and 380 nm restricted by using a rotating filter wheel as previously described (Lorenz et al., 1999; Han et al., 2010). Emitted fluorescence was detected at 510 nm (restricted using a bandpass filter) using a photomultiplier tube. The ratio of emitted fluorescence at 340 and 380 nm excitation wavelengths (F_340_/F_380_) was used to determine [Ca^2+^]_*cyt*_ based on a calibration equation described by Grynkiewicz et al. (1985); Todoroki-Ikeda et al. (2000), Han et al. (2010), and Sieck et al., 2019. Duplicate measurements of isometric [Ca^2+^]_*cyt*_ and force responses of ASM strips to ACh concentration (EC_50_) were digitized (1 kHz sampling rate) using LabChart8 (AD Instruments, Colorado Springs, CO, United States). The duplicate measurements were subsequently averaged for each trachea.

### Measurement of rMLC_20_ Phosphorylation in Response to ACh Stimulation

As previously described (Sieck et al., 2019), rMLC_20_ phosphorylation was detected using a Phos-tag^TM^ sodium dodecyl sulfate-polyacrylamide (SDS-PAGE) gel with Zn^2+^ (Takeya et al., 2008; Walsh et al., 2011; Sieck et al., 2019; Han et al., 2021) (Wako Chemicals Inc., Richmond, VA, United States) and standard western blotting. In each of the TNFα treated and control groups, one ASM strip was not exposed to ACh and was thus used to determine the baseline level of rMLC_20_ phosphorylation. The second ASM strip in each group was stimulated with ACh concentration (EC_50_) for 30 s, a time period previously found to induce maximum rMLC_20_ phosphorylation (Parkman et al., 2001). The ASM strips were then flash-frozen in 10% trichloroacetic acid/10 mM dithiothreitol in pre-chilled acetone. Electrophoresis of the Phos-tag^TM^ gels was performed at 20 mA for 1 h 50 min in running buffer (pH 7.4; 100 mM Tris-base, 100 mM 3-morpholinopropane-1-sulfonic acid, 0.1% SDS, 5 mM Sodium Bisulfate) at the room temperature. The gel was immersed in 25 mM Tris, 192 mM Glycine, 10% methanol (v/v) containing 10 mM EDTA to remove Zn^2+^ for 30 min and proteins were transferred to polyvinylidene difluoride membrane, which was fixed with 0.5% formaldehyde in PBS for 45 min (Takeya et al., 2008) and blocked with 5% dry milk in TBST. Using a standard western blotting technique, the membrane was incubated with primary (1:2000 dilution, rabbit polyclonal anti-rMLC_20_; sc-15370; Santa Cruz, CA, United States) and secondary antibody (1:10,000 dilution, goat anti-rabbit IgG-HRP conjugate; Santa Cruz, CA, United States). Unphosphorylated rMLC_20_ and phosphorylated rMLC_20_ (p-rMLC_20_) were detected by enhanced chemiluminescence (SuperSignal West Dura Extended Duration Substrate; Thermo Fisher Scientific, Rockford, IL, United States) and imaged on ChemiDoc MP Image System (Bio-Rad Laboratories, Hercules, CA, United States) and analyzed using an Image-Lab-Software (version 6.0.1, Bio-Rad Laboratories, Hercules, CA, United States) (Figure 5C).

**FIGURE 2:**
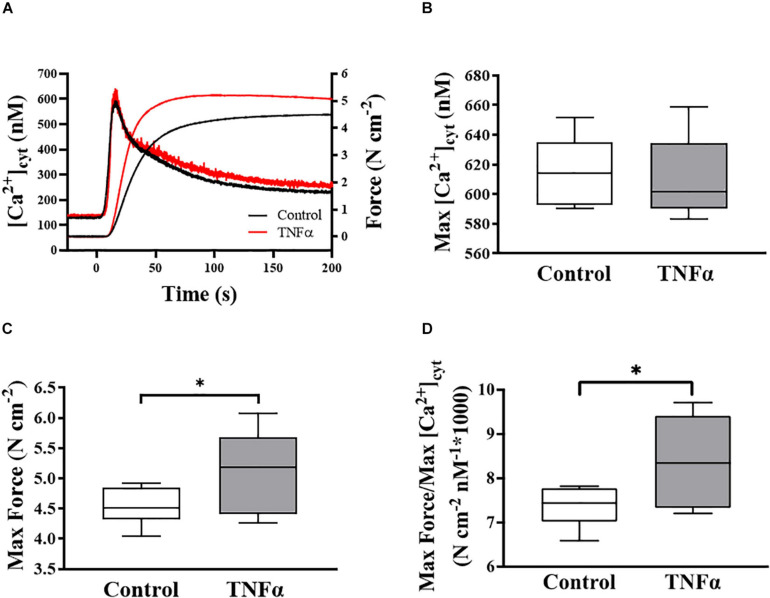
Representative tracings of simultaneous measurements of [Ca^2+^]_*cyt*_ and force responses of ASM to ACh stimulation (EC_50_) **(A)**. Maximum specific force **(C)** and static Ca^2+^ sensitivity of ASM significantly increased **(D)** with TNFα exposure, while there was no significant change in maximum [Ca^2+^]_*cyt*_ **(B)** (*p* > 0.05), compared to controls [summarized as medians and interquartile range (IQR)]. Data were analyzed using a one-way repeated measure ANOVA. Significance was considered at *p* < 0.05 [^∗^compared with TNFα; *n* = 6 (number of animals)].

**FIGURE 3:**
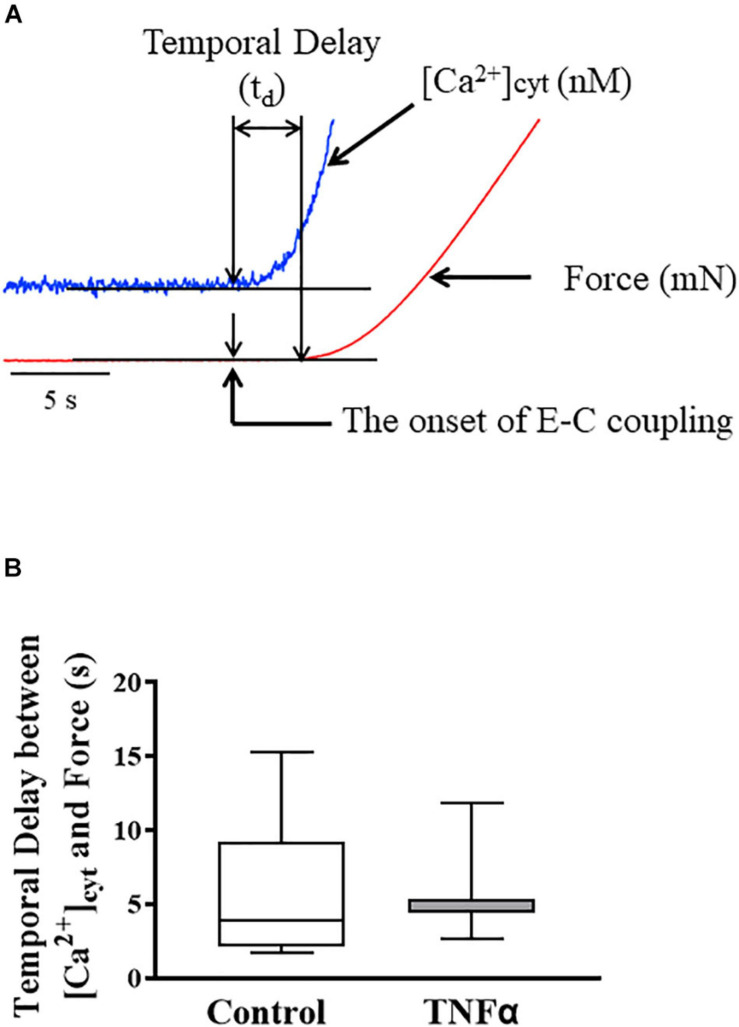
Representative tracings of [Ca^2+^]_*cyt*_ (blue) and force (red) response of ASM to ACh stimulation **(A)**. The temporal delay (t_*d*_) between the onset of [Ca^2+^]_*cyt*_ and force responses was not dependent on TNFα **(B)**. A one-way repeated measure ANOVA was used to analyze the results; *p* > 0.05, *n* = 6 (number of animals) and summarized as medians and interquartile range (IQR).

**FIGURE 4:**
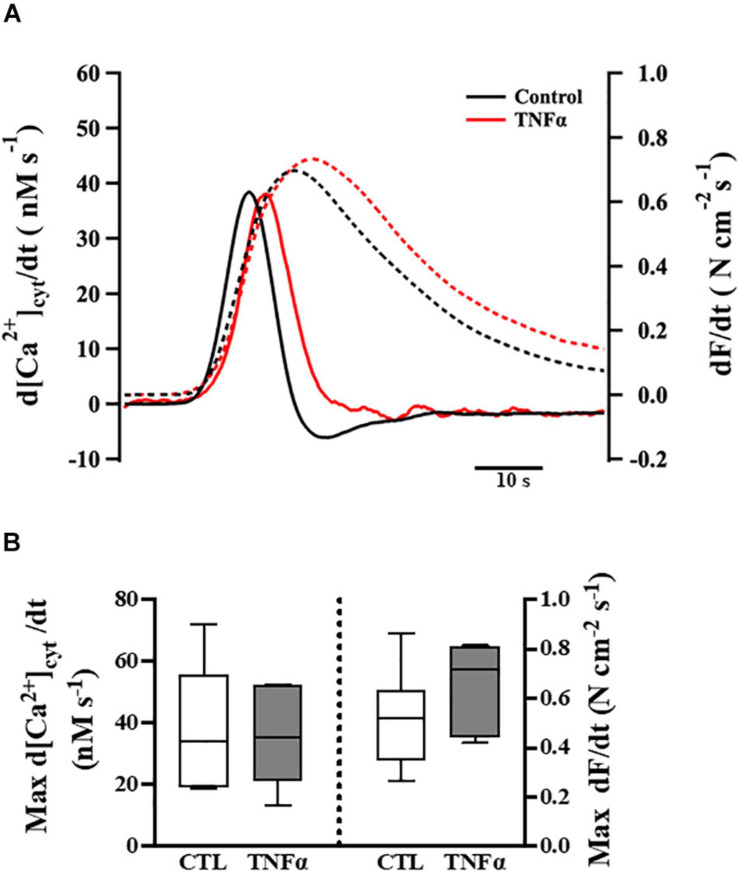
Tumor necrosis factor α exposure had no significant effects on the rates of rise of both the [Ca^2+^]_*cyt*_ and force responses of ASM to ACh stimulation (EC_50_), compared to controls. Representative tracings to show the first derivatives of [Ca^2+^]_*cyt*_ (solid lines) and force (dashed lines) responses to ACh stimulation (EC_50_) **(A)**. The maximum d[Ca^2+^]_*cyt*_/dt and maximum *d*Force/*d*t at ACh concentration (EC_50_) are summarized as medians and interquartile range (IQR) **(B)**. A one-way repeated measure ANOVA was used to analyze results; *p* > 0.05, *n* = 6 (number of animals).

**FIGURE 5:**
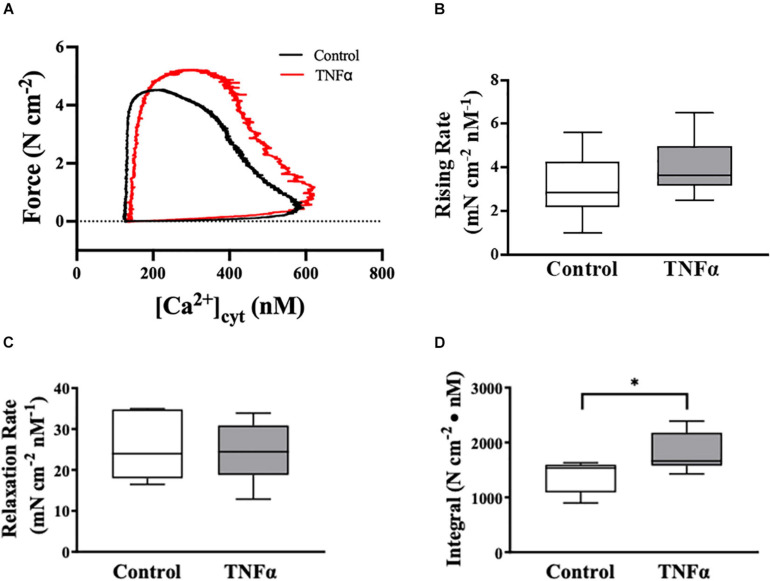
Tumor necrosis factor αsignificantly increased the integral of the rising phase of the phase-loop plots of ACh-induced [Ca^2+^]_*cyt*_ and force as compared to controls. Representative tracings of phase-loop plots at ACh concentration (EC_50_) are presented in **(A)**. Both rising rate and relaxation rate of the phase-loop plots in TNFα treated group were not different than untreated controls [*p* > 0.05, *n* = 6 (number of animals)]. **(B,C)** The integral of the rising phase of the phase-loop plots of ACh-induced [Ca^2+^]_*cyt*_ and force was summarized and found to increase significantly with TNFα [^∗^*p* < 0.05, *n* = 6 (number of animals)] **(D)**.

### Statistical Analysis

Four ASM strips were used from each animal (*n* = 6) for the analysis of [Ca^2+^]_*cyt*_ and force responses induced by ACh stimulation with (two strips) and without (two strips) 24-h exposure to TNFα. The two independent measurements of [Ca^2+^]_*cyt*_ and force responses to ACh were then averaged to provide a single measurement per treatment per animal. In a separate set of four ASM strips from each animal (*n* = 6), baseline (no ACh stimulation) and ACh-induced changes in rMLC_20_ phosphorylation were analyzed with (two strips – one baseline and one ACh stimulated) and without (two strips – one baseline and one ACh stimulated) 24-h exposure to TNFα. Results were analyzed using a Student *t*-test (JMP Pro software; JMP, RRID: SCR_014242). Data are summarized as means ± SD, with significance established at *p* < 0.05.

## Results

### Effects of TNFα on ACh-Induced [Ca^2+^]_*cyt*_ and Force Responses

In each of six animals (*n* = 6), the duplicate measurements of ASM [Ca^2+^]_*cyt*_ and force responses to ACh stimulation were comparable (<10% variance between the two measurements) and averaged to obtain a single value for summary analysis. In each ASM strip, ACh stimulation induced a biphasic [Ca^2+^]_*cyt*_ response reaching an initial peak by ∼5 s and then declining to a sustained plateau after ∼2–3 min. The isometric force response to ACh stimulation was delayed and slower with maximum specific force reached after ∼1–2 min. Compared to control (untreated) ASM strips, 24-h exposure to TNFα (100 ng/ml) increased the peak force response to ACh by ∼18% compared to control (Figures 2A,C; *p* < 0.05), whereas the peak [Ca^2+^]_*cyt*_ response to ACh was unaffected by TNFα (Figures 2A,B). The ratio of maximum specific force to maximum [Ca^2+^]_*cyt*_ increased by ∼18% in ASM strips exposed to 24-h TNFα (*p* < 0.01; Figure 2D). These results indicate that static Ca^2+^ sensitivity of force generation in ASM is increased by TNFα.

### TNFα Does Not Affect the Temporal Delay Between ACh-Induced [Ca^2+^]_*cyt*_ and Force Responses

In each ASM strip, [Ca^2+^]_*cyt*_ and isometric force responses to ACh stimulation were simultaneously recorded (Figure 3A). The ACh-induced elevation of [Ca^2+^]_*cyt*_ always preceded the force response by ∼3–5 s (Figure 3A). The fura-2 fluorescence signal of [Ca^2+^]_*cyt*_ was noisier affecting the signal to noise level and detection of the onset of the ACh-induced response. Yet, the signal to noise ratio of the [Ca^2+^]_*cyt*_ responses was less than 10%, which was sufficient to determine the onset of the [Ca^2+^]_*cyt*_ response within 10 ms with a 1 kHz sampling rate. The noise level of the mechanical force signal was much lower, and thus did not affect the detection of the onset of the ACh force response. The temporal delay between the onset of ACh-induced [Ca^2+^]_*cyt*_ and force responses was ∼4–5 s corresponding to the time to reach a peak [Ca^2+^]_*cyt*_ response. Importantly, compared to control (untreated) ASM strips, 24-h TNFα exposure had no impact on the temporal delay between the onset of ACh-induced [Ca^2+^]_*cyt*_ and force responses (Figure 3B).

### TNFα Does Not Affect the Rate of Rise of ACh-Induced [Ca^2+^]_*cyt*_ and Force Responses

The temporal characteristics of the ACh-induced [Ca^2+^]_*cyt*_ and force responses in ASM strips were determined by calculating the first derivatives of [Ca^2+^]_*cyt*_ and force responses (i.e., *d*[Ca^2+^]_*cyt*_/*d*t and *d*Force/*d*t) (Figure 4A). The maximum ACh-induced *d*[Ca^2+^]_*cyt*_/*d*t was ∼40 nM [Ca^2+^]_*cyt*_ s^–1^. Compared to control (untreated) ASM strips, 24-h TNFα exposure had no significant effect on the rate of rise in ACh-induced [Ca^2+^]_*cyt*_ response (Figure 4B). The maximum ACh-induced *d*Force/*d*t was much slower at ∼0.6 N cm^–2^ s^–1^. Although peak force was higher in TNFα treated ASM strips, the maximum *d*Force/*d*t was not different than untreated controls (Figure 4B).

### TNFα Impacts the Phase-Loop Plots of ACh-Induced [Ca^2+^]_*cyt*_ and Force Responses

The dynamic coupling of ACh-induced [Ca^2+^]_*cyt*_ and force responses in ASM strips was evaluated using phase-loop plots (Figure 5A). The ascending limb of the phase loop plot showed that little force developed as [Ca^2+^]_*cyt*_ increased to a peak value that was similar between 24-h TNFα treated and untreated control ASM strips. The rising rate of the phase-loop plots of ACh-induced [Ca^2+^]_*cyt*_ and force responses was similar between TNFα and control ASM strips (Figure 5B). Subsequently force increased as [Ca^2+^]_*cyt*_ decreased. Importantly, the ACh-induced force response in TNFα treated ASM strips was greater compared to untreated control ASM strips. The relaxation rate of the phase-loop plots of ACh-induced [Ca^2+^]_*cyt*_ and force responses was also similar between TNFα and control ASM strips (Figure 5C). To provide an overall assessment of the dynamic coupling of ACh-induced [Ca^2+^]_*cyt*_ and force responses, the integral of the phase-loop plots was calculated. Compared to control (untreated) ASM strips, TNFα significantly increased the integral of the phase-loop plot (*p* < 0.05, Figure 5D).

### TNFα Does Not Affect the Extent of ACh-Induced rMLC_20_ Phosphorylation

**FIGURE 6:**
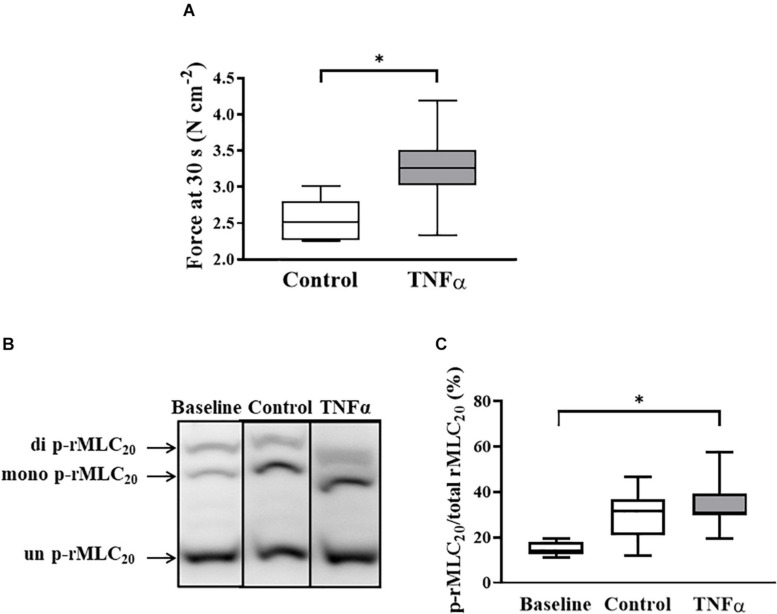
Tumor necrosis factor α had no significant effect on rMLC_20_ phosphorylation in ASM stimulated at ACh concentration (EC_50_) [*p* > 0.05, *n* = 6 (number of animals)] **(C)**, even though ACh stimulation significantly increased the extent of rMLC_20_ phosphorylation in both 24-h TNFα treated and untreated control ASM strips **(B,C)**, compared to the baseline (no ACh stimulation). However, force generated in ASM after 30 s at ACh stimulation (EC_50_) significantly increased with TNFα exposure, compared to controls [^∗^*p* < 0.05, *n* = 6 (number of animals)] **(A)**. Representative western blots to show phosphorylated (single- and double-p-rMLC_20_) and un-phosphorylated (un-p-rMLC_20_) rMLC_20_ using a modified Phos-tag^TM^ gels **(B)**.

In a separate set of ASM strips, the extent of rMLC_20_ phosphorylation (ratio of p-rMLC_20_ to total rMLC_20_) induced by 30 s ACh stimulation was examined in ASM strips using western blot analysis (Figure 6). The 30 s time period for ACh stimulation was selected as we have previously shown that the maximum extent of rMLC_20_ phosphorylation is reached by this time (Dogan et al., 2017). ACh stimulation significantly increased the extent of rMLC_20_ phosphorylation in both 24-h TNFα treated and untreated control ASM strips (Figures 6B,C), compared to the baseline (no ACh stimulation). Compared to control ASM strips, 24-h TNFα exposure had no significant effect on the extent of ACh-induced rMLC_20_ phosphorylation (Figures 6B,C), although the ACh-induced force generated by TNFα treated ASM strips after 30 s was significantly greater than that of control ASM strips (*p* < 0.05; Figure 6A). Thus, the increase in ACh-induced ASM force following TNFα exposure was unrelated to the extent of rMLC_20_ phosphorylation. These results are consistent with previous studies showing that TNFα increases ASM force generation due to increased contractile protein content (greater number of contractile units) and enhanced cytoskeletal remodeling (actin polymerization) resulting in increased tethering of contractile elements to the plasma membrane and force translation to the extracellular matrix (ECM) rather than changes in rMLC_20_ phosphorylation (Jones et al., 1999a; Zhang et al., 2015; Dogan et al., 2017; Sieck et al., 2019).

## Discussion

The present study demonstrates that in porcine ASM, 24-h TNFα exposure significantly increases the force response induced by ACh stimulation while not affecting the [Ca^2+^]_*cyt*_ response. Thus, by definition the Ca^2+^ sensitivity of force generation in ASM is increased by TNFα exposure. To further explore the impact of TNFα on the dynamic nature of Ca^2+^ sensitivity in ASM, the present study simultaneously measured [Ca^2+^]_*cyt*_ and force responses to ACh stimulation. The initial coupling of [Ca^2+^]_*cyt*_ and force responses involves the canonical signaling cascade that regulates rMLC_20_ phosphorylation and cross-bridge recruitment. An effect of TNFα on this regulatory signaling cascade would have been reflected by a reduced temporal delay in the shorter-term coupling of ACh-induced [Ca^2+^]_*cyt*_ and force responses. However, the results of the present study showed no effect of TNFα on the temporal delay between the onset of [Ca^2+^]_*cyt*_ and force responses or on the rate of rise in either parameter. Previous results from our lab indicated that 24-h TNFα exposure increases contractile protein concentration in ASM (Dogan et al., 2017; Sieck et al., 2019) and the tethering of actin to the cortical cytoskeleton (Sieck et al., 2019). The coupling between elevated [Ca^2+^]_*cyt*_, contractile unit recruitment and internal loading in ASM involves much longer time delays that are affected by TNFα exposure but independent of rMLC_20_ phosphorylation.

### Does the Canonical rMLC_20_ Phosphorylation Pathway Regulate Ca^2+^ Sensitivity in ASM?

It is commonly thought that an increase in Ca^2+^ sensitivity of force generation in ASM is mediated by increasing the extent of rMLC_20_ phosphorylation *via* agonist-induced activation of rho kinase (ROCK)-dependent inhibition of myosin light chain phosphatase (MLCP) (de Lanerolle and Paul, 1991; Jones et al., 1994a, 1999a; Somlyo and Somlyo, 1994; Karaki et al., 1997; Sieck et al., 1998; Todoroki-Ikeda et al., 2000; Sakamoto et al., 2003; Sakai et al., 2017; Alvarez-Santos et al., 2020). However, we and others previously reported that the extent of rMLC_20_ phosphorylation induced by ACh stimulation reaches a maximum after ∼30 s or less and does not change thereafter (Jones et al., 1999a; Mehta and Gunst, 1999; Zhang et al., 2015; Dogan et al., 2017; Sieck et al., 2019; Seow and An, 2020; Han et al., 2021). This timeframe does not match the time for force generation in ASM, which reaches a maximum only after 60–120 s. Moreover, the extent of rMLC_20_ phosphorylation does not change with increasing ACh concentration, even though force increases (Dogan et al., 2017; Sieck et al., 2019; Han et al., 2021). Thus, while [Ca^2+^]_*cyt*_-dependent rMLC_20_ phosphorylation does mediate cross-bridge recruitment and the onset of a force response in ASM, it does not appear to regulate the longer-term time course or amplitude of the force response. In the present study, even at the lower EC_50_ concentration of ACh (1.3 vs 2.6 μM in TNFα treated vs untreated control, respectively), the force response in TNFα treated ASM was greater while the extent of rMLC_20_ phosphorylation was unaffected. In agreement, there was also no effect of TNFα exposure on the temporal delay between the onset of [Ca^2+^]_*cyt*_ and force responses or on their rates of rise after ACh stimulation. These results indicate that neither the extent nor the rate of rMLC_20_ phosphorylation in ASM is affected by TNFα exposure. Thus, we conclude that ACh-dependent rMLC_20_ phosphorylation is not the primary mechanism through which Ca^2+^ sensitivity of force generation increases after 24-h TNFα exposure.

### Short-Term Dynamic Coupling of ACh-Induced [Ca^2+^]_*cyt*_ and Force Responses

In ASM, muscarinic stimulation induces a biphasic elevation of [Ca^2+^]_*cyt*_ with a peak level reach in ∼5 s followed by a slow decline to steady state after ∼120–180 s. The initial elevation of [Ca^2+^]_*cyt*_ in response to ACh stimulation is due to several sources of Ca^2+^ including inositol triphosphate receptor (IP_3_R) and ryanodine receptor (RyR) mediated sarcoplasmic reticulum (SR) Ca^2+^ release as well as extracellular Ca^2+^ influx *via* L-type, voltage-gated Ca^2+^ channels (Takuwa et al., 1987; Jones et al., 1994b). Both the IP_3_R and RyR channels are Ca^2+^ sensitive resulting in Ca^2+^-induced SR Ca^2+^ release (CICR) and a more rapid accelerating elevation of [Ca^2+^]_*cyt*_ (Kannan et al., 1997; Sieck et al., 1997; Prakash et al., 2000; Pabelick et al., 2001; Bai et al., 2009). ACh stimulation activates phospholipase C and IP_3_ production, and IP_3_ mediates the opening of IP_3_R channels in the SR. In contrast, there is no second messenger agonist to mediate the opening of RyR channels in the SR. Instead, with the initial elevation of [Ca^2+^]_*cyt*_ due to extracellular Ca^2+^ influx or IP_3_R mediated SR Ca^2+^ release, CICR mediates the opening of RyR channels in ASM. However, ACh-induced production of cyclic ADP ribose increases the sensitivity of RyR to CICR (Kannan et al., 1996; Hsuan et al., 1999; White et al., 2000; Deshpande et al., 2004). In porcine ASM, we previously showed that ACh stimulation induces localized constant amplitude [Ca^2+^]_*cyt*_ oscillations that propagate through the cell (Prakash et al., 1997; Pabelick et al., 1999; Sieck et al., 2001). The global elevation of [Ca^2+^]_*cyt*_ in response to muscarinic stimulation reflects the spatial-temporal integration of these propagating [Ca^2+^]_*cyt*_ oscillations (Prakash et al., 1997; Pabelick et al., 1999; Sieck et al., 2001). While the amplitude of localized [Ca^2+^]_*cyt*_ oscillations is constant, both [Ca^2+^]_*cyt*_ oscillation frequency and propagation velocity are increased by increasing ACh concentration. The time course of the spatial/temporal summation of ACh-induced [Ca^2+^]_*cyt*_ oscillations in ASM cells is ∼700 ms, roughly matching the short-term delay in rMLC_20_ phosphorylation and cross-bridge recruitment (Figure 1; Prakash et al., 1997; Pabelick et al., 1999; Sieck et al., 2001). Measurements of the time delay between the onset of [Ca^2+^]_*cyt*_ and force responses as well as the first derivatives of these responses (i.e., *d*[Ca^2+^]_*cyt*_/*d*t and *d*Force/*d*t) reflect the short-term dynamic coupling mediated by rMLC_20_ phosphorylation and cross-bridge recruitment (Figures 3A,B). Importantly, 24-h TNFα exposure did not affect these measures of short-term coupling of ACh-induced [Ca^2+^]_*cyt*_ and force responses.

### Long-Term Dynamic Coupling of ACh-Induced [Ca^2+^]_*cyt*_ and Force Responses

Unlike the [Ca^2+^]_*cyt*_ response, the ACh-induced force response in ASM is not biphasic but develops at a much slower rate reaching a plateau in force only after 60–120 s. As shown in Figure 1, short-term force generation in ASM depends on [Ca^2+^]_*cyt*_/calmodulin-mediated activation of MLCK and rMLC_20_ phosphorylation and cross-bridge recruitment. The phosphorylation of rMLC_20_ is also affected by MLCP and dephosphorylation; however, we and others have shown that the maximum level of ACh-induced rMLC_20_ phosphorylation in ASM is reached and sustained after ∼30 s or less, well before the plateau in ACh-induced force (Washabau et al., 1991; Dogan et al., 2017; Sieck et al., 2019; Han et al., 2021). Importantly, ACh-induced force generation in ASM increases by the addition of *de novo* contractile units due to [Ca^2+^]_*cyt*_-dependent polymerization of actin and myosin, and to the tethering of filamentous actin to the cortical cytoskeleton for force translation to the ECM (Figure 1; Gunst and Tang, 2000; Sieck et al., 2001; Murphy and Rembold, 2005; Ijpma et al., 2010, 2011; Sieck and Gransee, 2012; Delmotte et al., 2020; Seow and An, 2020; Wang et al., 2020). It can be assumed that by ∼30 s after ACh stimulation, when the maximum extent of rMLC_20_ phosphorylation is achieved, the recruitment of available cross-bridges does not account for additional force generation. Thus, from 30 to ∼120 s after ACh stimulation when ASM force plateaus, the addition of *de novo* contractile units or the tethering of actin filaments to the cortical cytoskeleton are the major contributors to force generation.

The cycling of cross-bridges in ASM is associated with ATP hydrolysis, and is dependent on load (Jones et al., 1999a; Delmotte et al., 2020). The tethering of actin filaments to the cortical cytoskeleton *via* dense bodies allows ASM force transmission to the ECM (Jones et al., 1999b; Mehta and Gunst, 1999; Gunst and Fredberg, 2003; Gunst et al., 2003; Gunst and Zhang, 2008; Zhang and Gunst, 2008; Chitano et al., 2017; Dogan et al., 2017; Tang, 2018; Seow and An, 2020; Wang et al., 2020). At the same time, the tethering of contractile units increases internal loading and slows cross-bridge cycling rate (Dogan et al., 2017; Sieck et al., 2019; Han et al., 2021). In permeabilized ASM strips maximally activated by Ca^2+^ (pCa 4.0), we simultaneously measured force and ATP hydrolysis rate using an NADH-linked fluorescence technique (Delmotte et al., 2020). The maximal increase in ATP hydrolysis rate occurred during the initial 30–60 s with the rMLC_20_ phosphorylation-mediated recruitment of cross-bridges and the *de novo* addition of contractile units. Thereafter, the increase in ATP hydrolysis rate slowed reflecting the tethering of contractile units to the cortical cytoskeleton and internal loading with peak ATP hydrolysis rate reached after ∼120 s (Delmotte et al., 2020). Thereafter, ATP hydrolysis rate decreased with further tethering of contractile units and increased internal loading until a plateau was reached after ∼300–400 s.

The polymerization of monomeric globular (G) actin to filamentous (F) actin is Ca^2+^ dependent and increases with ACh stimulation (Hirshman and Emala, 1999; Jones et al., 1999b; Mehta and Gunst, 1999; Sieck et al., 2019). Zhang et al. reported that ACh-induced actin polymerization in ASM is regulated by RhoA activation, which enhances the assembly of the adhesome within the cortical cytoskeleton (Zhang et al., 2012; Seow and An, 2020). In support, we found that the extent of actin polymerization and tethering to the cortical cytoskeleton increases with time and with ACh concentration (Brandenburg et al., 2019). Furthermore, inhibiting actin polymerization greatly reduces ASM force generation (Jones et al., 1999b; Mehta and Gunst, 1999; Sieck et al., 2019; Delmotte et al., 2020; Han et al., 2021), reflecting the disruption of both the contribution of additional contractile units and force translation due to the tethering of contractile elements to the cortical cytoskeleton.

### Integral of Phase-Loop Plots of [Ca^2+^]_*cyt*_ and Force Responses to ACh Stimulation

In the heart, phase-loop plots of [Ca^2+^]_*cyt*_ and force responses evoked by electrical stimulation have been used to assess Ca^2+^ sensitivity of cardiomyocytes based on the [Ca^2+^]_*cyt*_ at which 50% relaxation occurs (Schaible et al., 2016; Tveita et al., 2019). This assessment reflects the off-rate of Ca^2+^ binding to troponin-C (TnC) and thin filament regulation of cross-bridge attachment/detachment in cardiomyocytes. In contrast, the coupling of [Ca^2+^]_*cyt*_ and force responses in ASM is far more complex; however, phase-loop plot of ACh-induced [Ca^2+^]_*cyt*_ and force responses still provides valuable physiological insight. The rising phase of the phase-loop plot most likely reflects rMLC_20_ phosphorylation and cross-bridge recruitment, while the relaxation rate most likely reflects rMLC_20_ dephosphorylation. Importantly, both the rising phase and the relaxation rate of the phase-loop plots of ACh-induced [Ca^2+^]_*cyt*_ and force responses were not affected by 24-h TNFα exposure. In contrast, the integral of the ACh-induced [Ca^2+^]_*cyt*_ and force phase-loop plot increased significantly after 24-h TNFα.

## Conclusion

We conclude that 24-h TNFα exposure increases the Ca^2+^ sensitivity of ASM force generation through an effect on the long-term dynamic coupling of ACh-induced [Ca^2+^]_*cyt*_ and force responses. Our results indicate that the increase in Ca^2+^ sensitivity induced by TNFα exposure is not dependent on rMLC_20_ phosphorylation or dephosphorylation. Instead, TNFα exposure increases actin and myosin concentration in ASM leading to the recruitment of additional contractile units. In addition, TNFα exposure increases actin tethering to cortical cytoskeleton and force translation to the ECM.

## Data Availability Statement

The raw data supporting the conclusions of this article will be made available by the authors, without undue reservation.

## Ethics Statement

The animal study was reviewed and approved by the Institutional Animal Care and Use Committee (IACUC) Mayo Clinic.

## Author Contributions

Y-SH, PD, and GS contributed the conception and design of the study, analyzed the data, performed the statistical analysis, and wrote the manuscript. Y-SH collected the data. All authors approved the final version of the manuscript and agreed to be accountable for all aspects of the work in ensuring that questions related to the accuracy or integrity of any part of the work were appropriately investigated and resolved.

## Conflict of Interest

The authors declare that the research was conducted in the absence of any commercial or financial relationships that could be construed as a potential conflict of interest.

## Publisher’s Note

All claims expressed in this article are solely those of the authors and do not necessarily represent those of their affiliated organizations, or those of the publisher, the editors and the reviewers. Any product that may be evaluated in this article, or claim that may be made by its manufacturer, is not guaranteed or endorsed by the publisher.
